# Benefits of Applying Nanotechnologies to Hydrogels in Efficacy Tests in Osteoarthritis Models—A Systematic Review of Preclinical Studies

**DOI:** 10.3390/ijms23158236

**Published:** 2022-07-26

**Authors:** Chiara Delbaldo, Matilde Tschon, Lucia Martini, Milena Fini, Giorgia Codispoti

**Affiliations:** Complex Structure of Surgical Sciences and Technologies, IRCCS Istituto Ortopedico Rizzoli, Via di Barbiano 1/10, 40136 Bologna, Italy; chiara.delbaldo@ior.it (C.D.); lucia.martini@ior.it (L.M.); milena.fini@ior.it (M.F.); giorgia.codispoti@ior.it (G.C.)

**Keywords:** nanoparticles, hydrogel, osteoarthritis, in vitro and in vivo models

## Abstract

Osteoarthritis (OA) is a severe musculoskeletal disease with an increasing incidence in the worldwide population. Recent research has focused on the development of innovative strategies to prevent articular cartilage damage and slow down OA progression, and nanotechnologies applied to hydrogels have gained particular interest. The aim of this systematic review is to investigate the state of the art on preclinical in vitro and in vivo efficacy studies applying nanotechnologies to hydrogels in OA models to elucidate the benefits of their applications. Three databases were consulted for eligible papers. The inclusion criteria were in vitro and in vivo preclinical studies, using OA cells or OA animal models, and testing hydrogels and nanoparticles (NPs) over the last ten years. Data extraction and quality assessment were performed. Eleven papers were included. In vitro studies evidenced that NP-gels do not impact on cell viability and do not cause inflammation in OA cell phenotypes. In vivo research on rodents showed that these treatments could increase drug retention in joints, reducing inflammation and preventing articular cartilage damage. Nanotechnologies in preclinical efficacy tests are still new and require extensive studies and technical hits to determine the efficacy, safety, fate, and localization of NPs for translation into an effective therapy for OA patients.

## 1. Introduction

Osteoarthritis (OA) is the most common chronic musculoskeletal disease, affecting 250 million people worldwide [1], including 18% of women and 10% of men aged 60 or over. This pathology can affect multiple body joints, with a greater prevalence in knees, followed by hips, hands, and spines. An increase in the prevalence of OA means an increase in direct and indirect costs for the health systems and people affected, with a total cost of EUR 2.5 billion per year [2,3,4,5].

Many studies have defined OA as a pathological condition characterized by progressive joint damage, inflammation, and articular cartilage loss, and it is associated with different risk factors. Patient-related risk factors include age, sex, gender, ethnic differences, diet, sedentary lifestyle, and obesity, whereas joint-related risk factors are type of injury, malalignment, joint shape, and loading [6,7]. Among all risk factors, sex and gender could play a key role in diagnosis, prognosis, and personalized therapy, although, currently, the treatment is identical for both men and women [8,9]. The treatments for OA are divided into non-pharmacological, pharmacological, and interventional treatments [10]. During the early stages of the disease, the Osteoarthritis Research Society International (OARSI) guidelines suggest physical activity and weight loss, and pharmacological treatments, based on the administration of oral and topical non-steroidal anti-inflammatory drugs (NSAIDs), are recommended in the more advanced phases to treat pain [11]. For patients with knee OA and comorbidities that hamper the assumption of NSAIDs or non-responsive, intra-articular (i.a.) corticosteroid injections that reduce the production of inflammatory cytokines and pain are advised. Before total joint replacement [12], another therapeutic option is the viscosupplementation that consists of i.a. injections of products that can restore the viscoelasticity of synovial fluid, reduce pain (thanks to a mechanical pillow action), and exert a protective effect on chondrocytes. Many viscosupplementation products are synthesized as derivatives of natural or synthetic cross-linked hyaluronic acid and polysaccharides such as collagen, chitosan, gelatin, or synthetic polymeric hydrogels like poly-caprolactone, polyglycolides, and their copolymers [13,14]. Although several studies have showed the effectiveness and safety of these products, repeated administrations are required due to their rapid clearance and low retention in the joint cavity, thus encouraging research to evaluate new treatments [8,15].

Hydrogels are water-swollen, 3D materials that can be injected in a minimally invasive manner in the joint [16]. In OA, hydrogels are interesting materials because of their good biocompatibility and biodegradability, mechanical and lubricant functions, hydrophilicity (for swelling and hydration), and minimally invasive approach. From a research point of view, they can be used as scaffolds for orthobiologics such as cells and growth factors, or as local drug delivery systems. Their major drawbacks are their low retention time in the joint due to the highly demanding mechanical compression, harsh OA microenvironment, and lack of regenerative capacities [17,18,19].

Some of the aforementioned limits may be overcome by adding lipid-based and cationic nanoparticles (NPs), microparticles, or liposomes to hydrogel systems, with the multiple aims of increasing their residence time and therapeutic efficacy, prolonging the lubrication in the diseased joint, and promoting articular cartilage regeneration [20].

Nanotechnology has been developed to mimic the nanoscale dimension of cartilaginous ECM and stimulate cell adhesion, migration, and proliferation, as well as to serve as local carriers of molecules for controlling their release, simultaneously protecting them from the harsh OA microenvironment and increasing their residence time in the joint. The precise control of the NP’s structure, degradation, and safety issues represent their main limits [21,22,23] NPs, defined as structures that reach dimensions of up to hundreds of nanometers [24], can be embedded into a hydrogel before its gelation or entrapped by gel swelling after gel formation [25]. These NP-enriched gel (NP-gels) formulations are of increasing interest because they can be used as drug delivery systems for anti-inflammatory drugs in OA, allowing controlled and prolonged release thanks to a higher retention in the joint than hydrogels alone. Moreover, NPs allow the drugs to reach their targets more easily as they facilitate drug diffusion through the thickness of the cartilage [13,26]. The presence of NPs might improve the mechanical and lubrication properties of hydrogels [27]. These systems can also be used as scaffolds for cartilage regenerative purposes by loading cells such as bone marrow stromal cells, synovial mesenchymal stem cells, adipose-derived stem cells, and chondrocytes [13,28].

The development of combined systems in which nanotechnology would increase the chemico-physical, mechanical, and detectability properties of hydrogels (and, on the contrary, hydrogels would promote the biological regenerative properties of NPs) could address many mechanical and biological cues arising in OA, thus being extremely relevant in this scenario.

Since the field of research on NP-gels is quite recent, the rationale of this study was to deeply investigate the current state of the art. The aim of the present systematic review is to identify the recent literature on the efficacy profile of NP-gel systems and discuss the set up utilized for their preclinical in vitro and in vivo investigations for their final application in OA treatment.

## 2. Methods

### 2.1. Search Strategy

The present literature review involved a systematic search carried out according to the PRISMA statement in three electronic databases (PubMed, Scopus, and Web of Knowledge: www.pubmed.gov, www.scopus.com, and www.webofknowledge.com). The search was applied with the following keywords: “(hydrogel OR gel) AND (nanoparticle OR nanosuspension OR nanosheet OR nanoformulation OR nano) AND (osteoarthritis OR OA OR osteoarthrosis)”. The screening process and analysis were conducted separately by three independent observers (C.D., G.C., and M.T.). Firstly, the articles were screened by title and abstract using the following inclusion criteria: in vitro and in vivo preclinical studies using OA cells (from patients or animals or commercially available) or OA animal models, testing hydrogelswith NPs. Additional criteria were publications in the English language and publication in the period 1 January 2013–31 December 2021. Exclusion criteria were articles written in other languages and reviews, abstracts, full texts not available, editorials or conference proceedings, clinical studies, and reports in which the OA model, hydrogels, and/or NPs were absent. Secondly, the reference lists of the included papers were screened to obtain further studies. Thirdly, duplicates were removed. The included papers were grouped according to the test model used, whether in vitro or in vivo. Disagreements were resolved by discussion and, where resolution was not possible, a fourth reviewer was consulted (L.M.).

### 2.2. Data Extraction

The papers’ main characteristics were extracted by C.D. and G.C., including for both in vitro and in vivo studies of the NP-gel systems, the intended clinical application, the main assays with selected experimental times, the main findings, and the first author’s name with the year of publication. In addition, for in vitro studies, data about the cell source, phenotype, and used passage were extracted. For in vivo studies, the OA animal model with number, sex, and strain of animals, NP-gel administration route, and timing of delivery were collected. Data were checked for accuracy and completeness by a third author (M.T.) and disagreements were resolved by discussion, and where resolution was not possible, the fourth reviewer was consulted (L.M.). We did not contact any authors.

### 2.3. Risk of Bias Assessment

A quality assessment of the in vitro studies was not performed since there is no validated tool of assessment.

Regarding the in vivo studies, the SYRCLE tool for animal studies has been applied: it consists of a 10-item checklist [29]. A low, high, or unclear risk of bias was scored if items were reported, not reported, or unclearly reported, respectively. The assessment was performed by two independent authors (C.D. and M.T.). Any disagreement was resolved by consensus with a third reviewer (G.C.).

## 3. Results

### 3.1. Search Strategy

**Figure 1:**
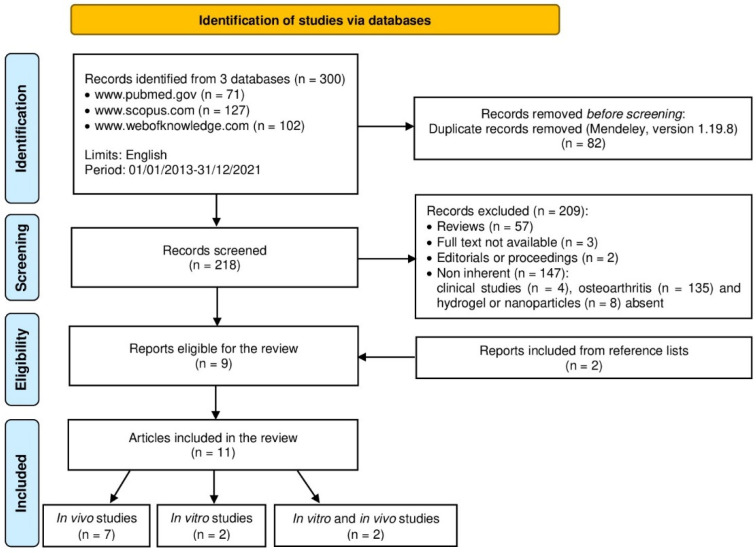
Search strategy according to the Preferred Reporting Items for Systematic Reviews and Meta-Analyses (PRISMA) guidelines.

An initial literature search was performed using the previously mentioned keywords, and 71 articles were retrieved using PubMed (www.pubmed.gov), 127 articles using Scopus (www.scopus.com), and 102 articles using Web of Knowledge (www.webofknowledge.com). Subsequently, the resulting references were submitted to a public reference manager (Mendeley 1.19.8, “www.mendeley.com”) to eliminate duplicate articles (n = 82). The remaining papers (n = 218) were screened for alignment with the inclusion criteria. Reviews (n = 57), full text not available (n = 3), editorials or proceedings (n = 2), and non-inherent papers including clinical studies (n = 4), papers with no osteoarthritis (n = 135), or those with no hydrogels or NPs (n = 8) were excluded. After screening, a total of nine articles were recognized as eligible for the review and, after examining the reference lists of these studies, two other papers were included. A total of eleven studies were definitely included in this review: two articles were in vitro studies, seven were in vivo, and two were both in vitro and in vivo (Figure 1).

### 3.2. In Vitro Preclinical Studies

**Figure 2:**
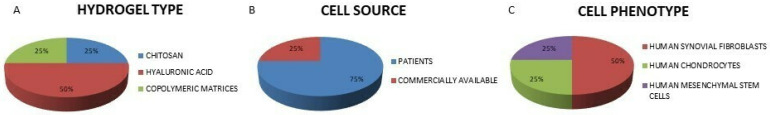
Diagrams with relative percentages of (**A**) type of hydrogel used; (**B**) sources of the cells; and (**C**) cell phenotype.

As shown in Table 1, most papers utilized hydrogels of natural origin or by combining naturally derived gel with synthetic polymers. Two papers out of four (50%) used hyaluronic acid hydrogels [30,31], whereas one paper (25%) used chitosan [32] and one (25%) used agar gel with a copolymeric matrix of PEGDA [33] (Figure 2A).

Different types of nanomaterials were tested: chitosan-kartogenin NPs were investigated by Kang et al. [32], synthetic PLA by Pradal et al. [30], and hyaluronic acid and hydroxyapatite by Maudens et al. and by Dua et al., respectively [31,33]. The NP dimension range was from 150 to 300 nm, as determined by scanning electron microscopy [31], dynamic light scattering [31,32], or nano zetasizer [30]. The tested NP-gel systems were developed in 50% of the studies as tridimensional scaffolds [31,33] and in the other 50% to locally deliver drugs in OA [30,32].

For the test systems, all studies utilized human primary cells, and 50% of the studies used synovial fibroblasts from OA patients undergoing prosthesis replacement procedures [30,31], while the remainder used hBMMSC and chondrocytes from OA patients (or commercially available sources) [32,33] (Figure 2B,C). The cells were used, after an expansion phase, between passages 3 and 10; the age range of patients was 54–76 years; and donors’ sex, smoking habits, and body mass index, and the presence of comorbidities, were not specified.

#### 3.2.1. Hyaluronic Acid Hydrogels

Most of the studies used hyaluronic acid hydrogels. Pradal et al. created a drug delivery system for i.a. injection based on PLA NPs with a size of 300 nm embedded in 0.6% hyaluronic acid in PBS [30]. Maudens et al. developed a scaffold for i.a. injections made with hyaluronic acid NPs at two different concentrations, 0.5% and 1%, encapsulated in a hyaluronic acid-pNiPAM hydrogel with a DBCO linker, and they obtained a physical cross-linking of the system [31].

In both studies, the cells used for the experiments were human synovial fibroblasts from OA patients, below passage 10 in the first case and at passage 8 in the second. A viability test was performed at 24 h after the treatment, showing that the NPs-gel system did not reduce cell survival.

#### 3.2.2. Chitosan Hydrogels

Kang et al. developed an i.a. drug delivery system that consisted of CHI-KGN fabricated by ionic gelation using tripolyphosphate. Human BMMSCs cultured in pellets (passages 3–5) from three patients undergoing hip prosthesis (with an age range of 54–72 years) were used. The authors also used chondrocytes at passage 3 from the articular cartilage of three patients undergoing knee arthroplasties (age range 59–65 years). The DNA quantity and GAG contents were evaluated on hBMMSCs, as well as the gene expression of *COLL I*, *COLL II*, *COLL X*, and *aggrecan* at 28 days. Histology was performed at 21 days. A viability test and IL-6 release assay were conducted on the chondrocytes at 7 days. The main findings showed that after treatment, there are no differences in the DNA quantity and gene expression of *COLL I* and *COLL X*, whereas the GAG content increased significantly when cells were exposed to CHI-KGN NPs versus those that were untreated. On the other hand, the gene expression of *COLL II* and *aggrecan* increased in the pellets exposed to CHI-KGN NPs for 21 days compared with untreated hBMMSCs. Safranin-O and Alcian Blue stainings that were associated with proteoglycan synthesis showed the greatest intensity in the pellets treated with CHI-KGN NPs. The chondrocytes treated with the CHI-KGN NPs below 100 nM showed normal cell proliferation profiles without a significant increase in IL-6 secretion, confirming that the NPs were not able to induce inflammation [32].

#### 3.2.3. Copolymeric Matrices

In the study by Dua et al., a two-layer engineered cartilage construct was developed. One layer contained human OA chondrocytes encapsulated in an agar gel via temperature-based gelation. Then, hBMMSCs suspended in a monomer solution of PEGDA, with and without HA NPs, were poured on top of the agar construct. The PEGDA solution subsequently underwent photopolymerization to form a gel structure. The HCOAs and hBMMSCs were commercially available products from OA patients used at passages 3 and 4, respectively. Live and dead assays were performed at different time points (1, 7, 14, and 28 days), showing a high cell viability after 28 days. Of note, the interfacial shear strength between the two layers was determined, showing a significantly higher shear strength in the HCOAs-based sample with HA NPs when compared to the corresponding group without HA. Moreover, the histological, EDS, and gene expression analyses confirmed the formation of a thin transition zone, made of calcium and phosphorus, between the hBMMSCs-derived engineered cartilage and the HCOAs-derived cartilage after 28 days of culture [33].

### 3.3. In Vivo Preclinical Studies

Four articles out of nine (44%) used chitosan hydrogels [32,34,35,36], whereas the remaining 56% used polymeric and copolymeric matrices [31,37,38,39,40]. The data were extracted and are reported in Table 2.

**Figure 3:**
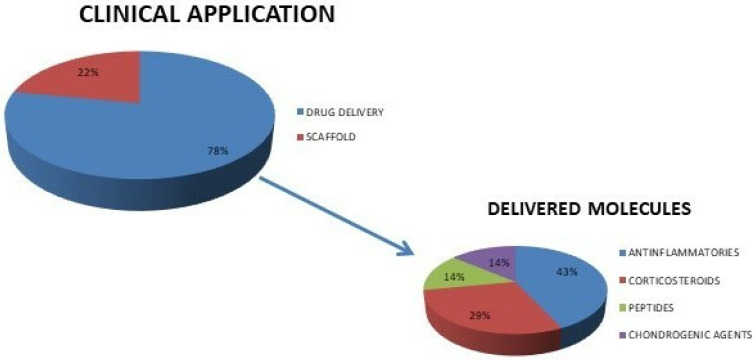
Diagrams of NPs-gel system clinical application and delivered molecules used in the included studies.

Regarding NPs, two studies used quercetin [34,35], two used PLGA with high affinity articular cartilage peptides [37] (or DEX-loaded [38]), one used chitosan-kartogenin [32], one used MSNs with colchicine [36], one used Hep/EPL [39], one used poly(organosphosphazes) with TCA [40], and one used hyaluronic acid [31]. The NPs’ dimensions varied in the range of between 140 and 387 nm. Their morphology was predominantly spherical, as reported in 44% of the works [32,34,35,36], whereas this information was not included in the remaining studies. Among the different clinical applications for which the NP-gel systems have been developed, six articles [32,34,35,36,37,40] out of nine used them for drug delivery, one used them for both drug delivery and for mechanical pillow function [38], one used them as scaffold [39], and one used them for viscosupplementation [31], as reported in Figure 3.

**Figure 4:**
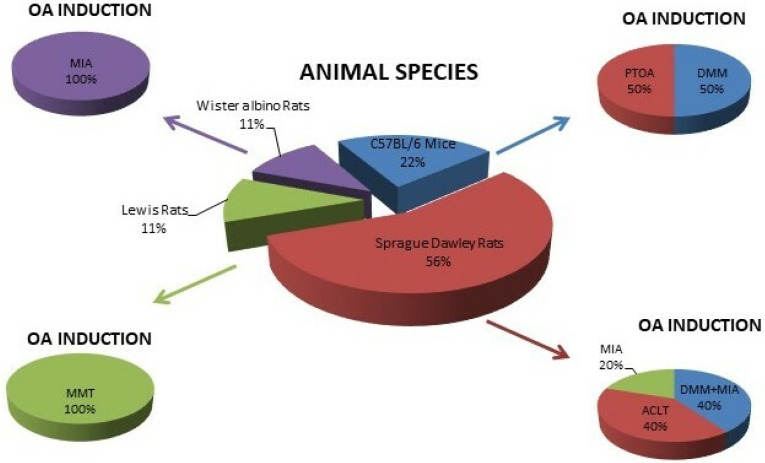
Diagrams of animal species and OA induction models adopted in the in vivo studies.

The drugs delivered with the NP-gel systems are: anti-inflammatory drugs administered via topical application [34,35,36], corticosteroids [38,40], and substances able to improve the chondrogenic differentiation of mesenchymal stem cells [32] or cartilage-, synoviocyte-, or integrin-binding peptides administered by i.a. injections [37]. Regarding the animal species and strains, all studies used rodents, as schematically depicted in Figure 4. Only male subjects were used by all authors, and the numbers of the animals for each experimental group ranged from a minimum of four to a maximum of nine, although an *a priori* power analysis has not been described.

#### 3.3.1. Mice

Two studies out of nine used C57BL/6 mice [31,38]. Maudens et al. developed a surgical OA model by unilateral DMM [31], whereas a PTOA model through daily cyclic compression loading on the tibia was adopted by Holyoak et al. [38] (Figure 4). These papers analyzed the viscosupplementation and drug delivery efficacy of two polymer-based hydrogels loaded with hyaluronic acid NPs and DEX-PLGA NPs, respectively. In DMM mice, the treatments were performed at days 7 and 35, whereas in PTOA mice, they started 48 h after the completion of the compression cycles [31,38]. Histological, microtomographic, and immunoenzymatic analyses showed that these treatments might attenuate the articular cartilage damage, even by reducing VEGF and proinflammatory cytokines expression [31,38]. Interestingly, Maudens et al. investigated the intravital persistence and microscopic fluorescence of NPs in the whole body, finding that hyaluronic acid NPs are located near the injection site within the synovial membrane and articular capsule for up to 2 months [31].

#### 3.3.2. Rats

The majority of studies (78%) used rats: five papers used the Sprague-Dawley strain [32,34,35,39,40], one utilized Lewis rats [37], and one adopted Wister albino rats [36] **(**Figure 4). In 44% of studies [32,37,39], OA was surgically induced by MMT or ACLT. In two other studies (28%), OA was chemically developed by i.a. injections of MIA [36,40]. Finally, two authors (28%) adopted and compared the therapeutic effects of the treatments in two different OA models, both by surgical and chemical induction (MMT and MIA) [34,35] (Figure 4).

Four papers out of seven (57%) selected the i.a. administration route both for drug delivery [32,37,40] and scaffold [39], and three studies (43%) adopted daily topical applications [34,35,36]. The timing of delivery was dependent on the type of OA induction. In rats with a surgical induction of OA (MMT and ACLT), treatments were intra-articularly delivered at 3, 6, and 9 weeks after surgery [32,37], whereas in the study performed using MIA, the treatment was delivered by an i.a. injection 1 week from induction [40]. In the articles using topical applications, quercetin NPs or colchicine loaded MSNs were tested [34,35,36]. Treatments started immediately after OA induction and continued for 21 and 42 days [34,35,36]. In these works, during the experiments, edema measurements were performed on animals every 7 days up to 70 days [34], whereas investigations on catabolic markers of inflammation on blood and histology on joints were conducted at 42 days [35]. The major findings showed that a higher dose of quercetin induced a significant reduction in edema volume after 14 days of daily topical administration in the MIA model. In the DMM model, significant decreases in edema measurements and inflammatory markers were obtained after 42 days, both locally at the joint and at the systemic level [34,35]. Mohamed et al. investigated the functional efficacy of hydrogels containing colchicine entrapped in MSNs delivered via transdermal daily applications on the knee. At the end of the treatment with the NPs-gel system, the locomotor activity showed an improvement compared to both the untreated control and the group treated with a drug-free hydrogel. Furthermore, the immunoenzymatic assays on blood displayed a reduction of the COX-2 and TNF-α in comparison with the untreated group. Finally, the histopathology on knee joints showed an OA-protective effect of the NPs-gel system. 

Concerning the NP-gel systems used by i.a. injective therapies, PLGA NPs with functionalizing peptides, Hep/EPL NPs in a copolymeric matrix [37,39], CHI-KGN NPs [32], and TCA NPs in a polymeric matrix [40] were tested. Three studies used surgical OA models (ACLT [32,39] and MMT [37]) to assess the efficacy of treatments and applied different tests: in vivo imaging by IVIS, in vivo localization of gels [37], and in vivo retention time in the joint [32]. These studies showed an increase in i.a. drug retention in the joints linked to the NPs-gel system [32,37] and an accumulation of gel in the synovial membrane [37]. Micro-CT [37], histology [32,37,39], and immunostaining morphometry [39] were performed, highlighting that NP-gel systems slow down OA progression [37].

Kang et al. and Tang et al. showed an improvement in OA treated joints measured by OARSI [32] and Mankin [39] scores, demonstrating an inhibition of ECM degradation and the prevention of collagen loss [39] 5 weeks post-treatments [32] and 8 weeks after surgery [39].

Finally, one study adopted chemical OA induction and performed x-ray, micro-CT, and histology 8 weeks after the injection of NPs-gel on MIA rats. The main findings demonstrated a reduction in inflammation and an improvement in anti-OA effects in every TePNs-treated group compared with the TCA solution groups. Furthermore, histology showed a morphological similarity of the TePNs hydrogel group with a normal cartilage. RT-PCR on blood was performed 1, 4, and 8 weeks after treatment. In the group treated with a TePNs hydrogel containing the highest concentration of TCA, 8 weeks after treatment, a reduction in pro-inflammatory cytokines levels and an increase in anti-inflammatory ones were shown [40].

#### 3.3.3. Risk of Bias Assessment

**Figure 5:**
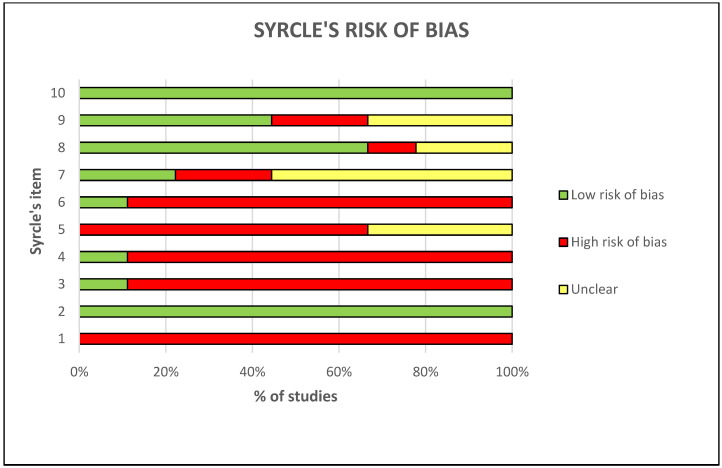
Each in vivo paper has been evaluated for the risk of bias by applying the SYRCLE’s tool [29]. For each of the 10 items, the frequency % of the low risk of bias (green bar), high risk of bias (red bar), and unclear risk of bias (yellow bar) is reported.

The risk of bias assessment is shown in Figure 5. The results of SYRCLE showed a high risk of bias in most of the items. In particular, at items 1 “sequence generation”, 3 “allocation concealment”, 4 “random housing”, 5 “blinding”, 6 “random outcome assessment”, the high risk of bias reached frequencies of 100%, 89%, 89%, 67%, and 89%, respectively. There was a low risk of bias at item 2 “baseline characteristics”, 8 “incomplete outcome data”, 9 “selecting reporting bias”, and 10 “other sources of bias”, with frequencies of 100%, 67%, 44%, and 100%, respectively. The remaining item 7 “blinding” presented a 56% of unclear risk of bias.

## 4. Discussion

The purpose of this systematic review was to analyze the state of the art on NP-gel systems, their properties, and in vitro and in vivo efficacy on cells and animal models for OA treatment. Highly hydrated hydrogels are biomaterials attracting interest in regenerative medicine owing to their mechanical and lubricating features and ECM biomimetic properties, and for their use as scaffold for tissue engineering approaches to create a suitable microenvironment to sustain cell proliferation or to deliver drugs and growth factors. Different properties, for instance, elasticity, adhesiveness, and mechanical features, render hydrogels eligible to entrap cells or drugs and deliver them in environments difficult to reach, such as the joint cavity [41]. The presence of NPs can ameliorate the lubrication and mechanical properties of hydrogels; moreover, they allow drugs to reach their targets more easily and enhance the retention time into the joint compared to the use of hydrogels alone. 

Regarding in vitro studies, NP-gel systems consisting of natural [30,32] or combined natural–synthetic hydrogels [31,33] containing NPs with different sizes of up to 400 nm are used as scaffolds and for drug delivery. All the studies used primary OA cells, mostly from OA patients, as synovial fibroblasts [30,31], chondrocytes, and mesenchymal stem cells [32], or they used commercially available cells [33]. However, other cell types such as macrophages can be utilized as in vitro OA models to recreate the inflammatory OA microenvironment and allow researchers to test the efficacy of new treatments that may inhibit the expression of proinflammatory molecules [42]. Thanks to their phagocytic nature, macrophages can also be relevant to investigate the NPs’ fates [43]. 

Concerning patient-derived cells, many other factors can be discussed, such as sex, gender, harmful lifestyle, and the presence of patient comorbidities that could affect the cellular phenotype [44,45]. In addition, the baseline characteristics of the OA phenotypes of cells were not reported—cells were even used at passages 8–10 without the maintenance of OA stimuli in cultures [30,31] or the determination of the differentiation status. For the maintenance of the OA stimuli, many different in vitro conditions could be set up to more closely reproduce the in vivo situation, and synovial fluid from OA patients and proinflammatory cytokines such as TNF-α or IL-1β, alone or in association, can be added to a culture medium to develop a catabolic phenotype [46,47]. Chemical agents, such as MIA or *tert*-butyl hydroperoxide, or mechanical stimuli can also promote OA pathway activation. By reproducing in vitro platforms of disease hallmarks, the screening of new therapeutics is allowed and realized in conditions that more closely represent the pathology [48]. Most authors used monolayer cell cultures, commonly used for in vitro assays, whereas some authors set up 3D cultures by seeding cells onto a solid scaffold or culturing in micro-masses [32,33]. Different models can be adopted to mimic in vitro human OA microenvironments, such as 2D co-cultures to investigate cell–cell interactions and 3D culture models, which are more representative of the in vivo joint architecture. Two-dimensional co-cultures involve many cell types grown in separate layers in a common microenvironment to investigate their interactions and the release of paracrine factors that can promote OA progression or improvement. Three-dimensional culture models include scaffold-based or scaffold-free approaches: the first uses a physical support such as a hydrogel for cell–cell, cell–ECM, and intercellular interactions, whereas a 3D culture based on a scaffold-free approach adopts high-density pellet cultures to study MSCs differentiation. In addition, explant and organ models can reproduce a condition closely related to a human one: they are based on single cultures of explants, such as chondral or osteochondral explant cultures, or co-cultures of different joint tissues, e.g., articular cartilage fragments with synovial membranes. Finally, culture conditions could be tailored to create a controlled OA microenvironment and promote cell–cell interactions, for example, bioreactors can be utilized to produce dynamic cultures, monitor the cellular parameters, and apply biomechanical forces. Recent advances in technological innovations have led to smart and precise biomaterial-cell deposition through 3D bioprinting to manufacture biological and clinically relevant constructs. Microfluidic devices consisting of a serial building of in vitro multi-tissue models within a microchip platform, able to precisely control the microenvironment and tune up mechanical and/or biochemical stimuli, could be relevant in the study of OA [48,49].

Ultrastructural tests to determine the safety and potential genotoxicity of NPs would be desirable. According to ISO 10993-22 (ISO/TR 10993-22:2017 Biological evaluation of medical devices, Part 22: Guidance on Nanomaterials), nano-objects can pass through the nuclear membrane and interact with DNA and nuclear proteins, leading to DNA lesions, affecting chromosome segregation, and causing oxidative stress. Different in vitro genotoxicity tests are suggested by selecting an integrated approach that comprises both mammalian and prokaryotic cell systems. Moreover, being in the -omic era, high throughput -omic analysis could add significant value to preclinical studies. 

In vivo studies have developed NP-gel systems for drug delivery [32,34,35,36,37,38,40], scaffolding [39], and viscosupplementation [31,38]. Some NP-gel systems were synthetized for the delivery of corticosteroids as DEX and TCA [38], chondrogenic agents as KGN [32], and anti-inflammatory molecules as quercetin [34,35] and colchicine [36]. KGN is a well-known small molecule with chondroprotective effects and is able to promote the differentiation of mesenchymal stem cells into chondrocytes, as showed in preclinical studies on OA animal models [50,51]. Quercetin is a flavonoid used for its anti-inflammatory, analgesic, and antioxidant effects, but its low bioavailability and solubility may be improved by encapsulating it in NP-gel systems [52]. Colchicine is another plant-extracted anti-inflammatory drug whose beneficial effects on OA knee patients have been widely demonstrated in clinical studies, showing its ability to reduce inflammation signs and pain and to improve knee joint function when administered in addition to conventional OA treatments [53]. 

The examined papers performed preclinical efficacy experiments on rodent OA models (both mice and rats). In vivo studies adopting medium and large OA animal models, such as rabbits, sheep, and goats, which are a commonly used animal models for OA surgical development (as suggested by OARSI guidelines), were not retrieved in the literature [54,55]. Furthermore, all the studies included in this review performed experiments on male subjects, although sex and gender affect OA onset and progression in both humans and in animals [8,9]. This is due to anatomical, physiological, mechanical, hormonal variations, and molecular aspects, such as different immune-inflammatory responses [56,57,58]. Moreover, even NPs distribution, accumulation, degradation rates, and toxicokinetic profiles are gender-related [59,60]. Some studies reported that male rats and mice show a higher OA incidence and severity compared to females, both in spontaneous and surgically induced OA models [61,62,63]. Indeed, chemically induced OA female rats exhibit a greater vulnerability to chronic pain than males [64]. These data suggest the importance of including both sexes in preclinical studies.

Regarding the performed analyses, histological and histomorphometrical analyses were the gold standard [31,35,36,40]. Many authors used in vivo non-invasive techniques repeated at different time points [31,32,34,36,37] that were compliant with the ethical requirements of the 3Rs principle (replacement, reduction, and refinement), reducing animal numbers, improving animal welfare, and avoiding, when possible, animal euthanasia [65,66]. 

Although limited to the last 10 years, the findings of the present review show that the preclinical research focussed on NP-systems efficacy testing is quite far for their clinical translatability. In the clinical setting, nanotechnologies for OA are still under development, with very few clinical experiments. A search in the PubMed database and the Clinicaltrials register highlighted that NPs are generally tested as nano-based emulsions or formulations to deliver analgesic NSAIDs or anti-inflammatory gold nanoparticles by topical and oral applications, and these clinical studies, mostly of phases one and two, demonstrated safety and efficacy with significantly improved pain, stiffness, and physical function in patients affected by OA [67,68,69,70,71,72,73,74,75] (NCT00484120, NCT05347602).

## 5. Conclusions

OA is a chronic and highly impactful inflammatory disease affecting an increasing population. Due to the complexity of the etiology and pathogenesis of OA, the efficacy of a single therapy seems to be overcome in favour of multi-composite treatments that could address many cues that arise in OA, which are tailored to patients. The research in nanomedicine is exponentially increasing, although, to date, the main limit is the translation of NP-gels from bench to bedside. To reach the clinical standards, biosafety issues should be checked by a multistep preclinical approach: it should comprise the complete chemico-physical characterization of the system, including its biomechanical, lubricating, and degradation properties, in vitro cytotoxicity testing with particular emphasis on macrophage responses to NPs, and in vivo safety and efficacy tests. Moreover, some issues related to the low reproducibility of the manufacturing processes, the stability of NPs (even in wet conditions), and the determination of NPs’ fates with detecting systems sensible enough to control NPs’ distribution in the whole body should be solved.

The combination of hydrogels with NPs aims to combine the already-confirmed benefits of the former with the innovation of the latter to simultaneously address the problems of joint lubrication, mechanical competence, chondroprotective effects, drug delivery, and residence time. Our review evidenced that preclinical research is still at the beginning and requires extensive studies and technical hits to determine the efficacy, safety, fate, and localization of NPs, both in vitro and in vivo, with the ultimate aim of translating into an effective therapy for OA patients.

## Figures and Tables

**Table 1:** Summary of the in vitro preclinical studies.

Hydrogel Nanoparticles System	Clinical Application	Cell Phenotype	Cell Source	Experimental Analyses (Experimental Times)	Main Findings	Ref.
Chitosan-kartogenin NPs (size 150 ± 39 nm, spherical)	Drug delivery (kartogenin)	hBMMSCs from bone marrow cultured in pellets (passages 3–5) and chondrocytes from articular cartilage (passage 3)	hBMMSCs: 3 patients undergoing hip prosthesis (age range: 54–72 years); chondrocytes: 3 patients undergoing knee arthroplasties (age range: 59–65 years)	hBMMSCs: DNA, GAG measure, and RT-PCR for *COLL I*, *COLL II*, *COLL X*, *aggrecan* (28 days), and histology (21 days); chondrocytes: MTT and IL-6 release (7 days)	hBMMSCs: no differences in DNA quantity, *COLL I*, and *COLL X* expression; increase in GAG contents, *COLL II*, and aggrecan; Safranin-O/Alcian Blue staining; chondrocytes: normal cell proliferation and no increase in IL-6 after treatment	Kang 2014 [32]
PLA nanoparticles (size 300 nm) in 0.6% hyaluronic acid	Drug delivery (DiD fluorescent stain)	Human synovial fibroblasts (below passage 10)	1 patient undergoing joint replacement (76 years)	Cell viability: MTT (24 h)	No reduction in cell viability after treatment	Pradal 2016 [30]
Hyaluronic acid nanoparticles (size 203–261 nm) 1% and 0.5% in hyaluronic acid-DBCO linker-pNiPAM	Scaffold	Human synovial fibroblasts isolated from synovial tissue (passage 8)	Synovial fibroblasts from 1 OA patient	Cell viability: MTT (24 h)	No reduction in cell viability after treatment	Maudens 2017 [31]
HA NPsin two-layer constructs: HCOAs in agar gel, hBMMSCs in PEGDA	Scaffold	HCOAs from cell applications (passage 4), hBMMSCs from Science Cell, Carlsbad, CA (passage 3)	Cells commercially available from OA patients	Cell viability: live and dead assay (1, 7, 14, and 28 days); mechanical testing: shear strength; histology and EDS: Von-Kossa and Alcian Blue stains (1 and 28 days); RT-PCR for *aggrecan*, *SOX9*, *COLL II*, *MMP-13*, *RUNX2*, *COLL X*, *COLL I*, and *osteocalcin* (after 28 days of culture)	Cell viability: ~86% viability of the HCOAs after 28 days; mechanical testing: higher shear strength in the NPs-gel system; histology and EDS: formation of a thin transition zone made of calcium and phosphorus; RT-PCR: lower expression of *COLL I*; maintaining expression of *aggrecan*, *SOX9*, and *COLL II*; no expression of *MMP-13*, *RUNX2*, and *COLL X*	Dua 2016 [33]

Abbreviations: *COLL I* = collagen type I; *COLL II* = collagen type II; *COLL X* = collagen type X; DBCO = dibenzocyclooctyne; DiD = (1,10-dioctadecyl-3,3,3′,3′-tetramethylindodicarbocyanine, 4-chlorobenzenesulfonate salt); HA = hydroxyapatite; EDS = energy-dispersive X-ray spectroscopy; GAG = glycosaminoglycans; hBMMSCs = human bone marrow-derived mesenchymal stem cells; HCOAs = human chondrocytes-osteoarthritic; IL-6 = interleukin 6; MMP-13 = matrix metallopeptidase 13; MTT = 3-(4,5-dimethylthiazol-2-yl)-2,5-diphenyl-2H-tetrazolium bromide; NPs = nanoparticles; PEGDA = poly (ethylene glycol) diacrylate; PLA = poly(D,L)-lactide; OA = osteoarthritis; pNiPAM = azide-terminated poly(Nisopropylacrylamide); RT-PCR = real time-polymerase chain reaction; RUNX2 = runt-related transcription factor 2; and SOX9 = SRY-box transcription factor 9.

**Table 2:** Summary of the in vivo preclinical studies.

Hydrogel Nanoparticles System	Clinical Application	Animal Model of OA (Number, Sex and Strain)	Administration Route and Timing of Delivery	Experimental Analyses	Main Findings	Experimental Times	Ref.
Quercetin NPs (size 212–242 nm, spherical) in chitosan	Drug delivery (3 doses of quercetin)	DMM and 3 mg/0.05 mL MIA in rats (5 male Sprague-Dawley rats for each group)	Topical application for 42 consecutive days	Edema volume measurements	MIA model: at day 14, significant differences using the higher dose of quercetin; DMM model: at day 42, significant differences using the higher dose of quercetin	7, 14, 21, 28, 35, 42, 49, 56, 63 and 70 days after OA induction	Karliana 2019 [34]
PLGA NPs (size 338 ± 91 nm) in PEG-4MAL macromer reacted with cartilage-(WYR), synoviocyte-(HAP-1), or integrin-(RGD) binding peptides	Drug delivery (cartilage-(WYR), synoviocyte-(HAP-1), or integrin-(RGD) binding peptides)	Unilateral MMT in rats (9 male Lewis rats for each group)	i.a injections 21 days after MMT	IVIS; micro-CT (volume, roughness and osteophyte volume); histology; in vivo localization of NP-gels	IVIS: NP-gel system increased in vivo intra-articular retention; micro-CT: WYR- and HAP-1 gels did not affect cartilage and OA progression; histology: no cartilage damage and synovial membrane thickening; in vivo localization: gel accumulation in the synovial membrane	IVIS: before and after 1, 3, 5, 7, 10, 13, 16, 19, and 26 days post-treatment; micro-CT: day 26; histology: at days 14 and 26	Mancipe Castro 2020 [37]
Chitosan-kartogenin NPs (size 150 ± 39 nm, spherical)	Drug delivery (kartogenin)	Bilateral ACLT in rats (8 male Sprague Dawley rats for each group)	Two i.a. applications after 42 and 63 days after ACLT	In vivo retention time; histology (OARSI score)	In vivo retention time: NPs showed long retention in the OA joint; histology: lower OARSI score in treated joints	In vivo retention time: 2, 4, 7, 14, and 24 days after treatment; histology: 35 days post-treatment	Kang 2014 [32]
Quercetin NPs (size 212.2 nm, spherical) in lecithin-chitosan	Drug delivery (3 doses of quercetin)	DMM and 3 mg/0.05 mL MIA in rats (5 male Sprague-Dawley rats for each group)	Topical application for 42 consecutive days	Histology; immunoenzymatic assays on blood (IL-1β, MMP-9, MMP-13, and ADAMTS5)	Improved histology; immunoenzymatic assays: all doses decreased IL-1β, MMP-9, MMP-13, and ADAMTS5 levels	Histology and immunoenzymatic assays on blood: 42 days after treatment	Permatasari 2019 [35]
DEX-loaded PLGA NPs (size 203 ± 7 nm) in PEG-4MAL macromers	Drug delivery (DEX) and mechanical pillow function	PTOA in mice by daily cyclic loading compression (9 N) on tibia for 42 days (5 male C57BL/6 mice for each group)	i.a. injections 48 h after compression	Histology (OARSI score, osteophyte dimensions)	NP-gel attenuated load-induced cartilage damage and osteophyte size	14 days after treatment	Holyoak 2019 [38]
Colchicine-loaded MSNs (size 167.1 ± 51.36 nm, spherical) in carboxyethyl chitosan and oxidized pullulan	Drug delivery (colchicine)	Unilateral 3 mg/joint MIA in rats (8 male Wister albino rats for each group)	Daily topical application of transdermal patches for 21 days (drug dose: 5 mg/kg/day)	Locomotor activity; immunoenzymatic assays on blood (TNF-α and COX-2); histology	Locomotor activity: NP-gel increased locomotor activity; immunoenzymatic assays: NP-gel reduced serum level of TNF-α and COX-2; histology: protective effects of NP-gel	Locomotor activity: 7 days before experiments; immunoenzymatic assays and histopathology: 21 days after MIA	Mohamed 2020 [36]
Hep/EPL NPs (size 387.81 ± 65.16 nm) dispersed in human PL and encapsulated in thermosensitive PLEL hydrogel	Scaffold	Bilateral ACLT in rats (4 Sprague-Dawley rats for each group)	Single i.a. injection	Histology and immunostaining (COLL II, MMP-13, and CD68, Mankin and synovitis scores)	Histology and immunostaining: NP-gel showed lower Mankin scores and better synovitis; immunostaining: treatment with NP-gel inhibited ECM degradation and prevented collagen loss	56 days after ACLT	Tang 2021 [39]
Poly (organosphosphazenes) NPs (size 140 ± 5 nm) encapsulated in polymeric hydrogel system	Drug delivery (3 doses of TCA)	0.5 mg/50 μL MIA in rats (6 male Sprague Dawley rats for each group)	i.a. injections of 0.3 mL, TePN solutions 7 days after OA induction	X-ray; microCT (distance of destroyed cartilages); histology; RT-PCR on blood (*MMP-3*, *MMP-13*, *IL-6*, *TNF-α*, *IL-4*, *IL-10*, and *IL-13*)	X-ray, histology, and micro-CT: NP-gel showed significant improvement in anti-OA effects; RT PCR: decrease in *MMP-3*, *MMP-13*, *IL-6*, and *TNF-α* levels, increase in *IL-4*, *IL-10*, and *IL-13*, in NP-gel	X-ray, micro-CT. and histology: 56 days after treatment; RT PCR: at days 7, 28, and 56	Seo 2021 [40]
HA nano (size 203–261, 377–435 by SEM) in DBCO linker-pNiPAM	Visco-supplementation	Unilateral DMM in mice (6-week-old male C57BL/6 mice, 7 for each group)	i.a. injections on days 7 and 35 after OA surgery	Intravital fluorescence and microscopic fluorescence; micro-CT (for medial/lateral tibial epiphysis thickness); histology (OARSI score); iImmunoenzymatic assays on blood (IL-1β, TNF-α, and VEGF)	Intravital and microscopic fluorescence: the residence time of HA nano exceeded 21 days near the injection site; micro-CT: NP-gel induced higher epiphysis thickness; histology: improved OARSI; immunoenzymatic assays on blood: NP-gel inhibited VEGF and reduced IL-1β and TNF-α	Histology, micro-CT, microscopic fluorescence, and blood analyses: day 63 after OA induction; intravital fluorescence: at days 0, 1, 7, and 21 after i.a. treatment	Maudens 2017 [31]

Abbreviations: ACLT = anterior cruciate ligament transection; ADAMTS5 = a disintegrin and metalloproteinase with thrombospondin motifs; COLL II = collagen type II; COX-2 = ciclooxigenase 2; DBCO = dibenzocyclooctyne; DEX = dexamethasone; DMM = destabilization of the medial meniscus; DTT = non-degradable dithiothreitol; ECM = extracellular matrix; Hep/EPL = heparin/ε-poly-l-lysine; HA nano = hyaluronic acid nanoparticles; HAP-1 = SFHQFARATLAS peptide; i.a. = intra-articular; IL-1β = interleukin 1β; IL-6 = interleukin 6; IL-4 = interleukin 4; IL-10 = interleukin 10; IL-13 = interleukin 13; IVIS = in vivo imaging; MIA = mono iodoacetate; micro-CT = micro computed tomography; MMT = medial meniscus transection; MMP-3 = matrix metallopeptidase 3; MMP-9 = matrix metallopeptidase 9; MMP-13 = matrix metallopeptidase 13; MSNs = mesoporous silica nanoparticles; NPs = nanoparticles; OA = osteoarthritis; OARSI = Osteoarthritis Research Society International; PEG-4MAL = 4-arm-poly(ethylene glycol)-maleimide; PL = platelet lysate; PLEL = poly(D,L-lactide)-poly(ethyleneglycol)-poly(D,L-lactide); PLGA = poly(lactic-coglycolic) acid; pNiPAM= poly(N-isopropylacrylamide); PTOA = post traumatic osteoarthritis; RGD = arginine, glycine, and aspartic acid peptide; RT-PCR = real time-polymerase chain reaction; SEM = scanning electron microscopy; TCA = triamcinolone acetonide; TePN = TCA-encapsulated polymeric nanoparticles; TNF-α = tumor necrosis factor α; VEGF = vascular endothelial growth factor; VPM = MMP degradable GCRDVPMSMRGGDRCG peptides; and WYR = WYRGRL peptide.

## Data Availability

Not applicable.

## Abbreviations

ACLT	Anterior cruciate ligament transection
CHI-KGN	Chitosan-kartogenin
COLL I	Collagen type I
COLL II	Collagen type II
COLL X	Collagen type X
COX-2	Cyclooxygenase 2
DBCO	Dibenzocyclooctyne
DEX	Dexamethasone
DMM	Medial meniscus destabilization
ECM	Extracellular matrix
EDS	Energy-dispersive X-ray spectroscopy
GAG	Glycosaminoglycan
HA	Hydroxyapatite
hBMMSC	Human bone marrow mesenchymal stem cells
HCOAs	Human osteoarthritic chondrocytes
Hep/EPL	Heparin/ε-poly-L-lysine
i.a.	Intra-articular
IL-1β	Interleukin 1β
IL-6	Interleukin 6
IVIS	In vivo imaging
KGN	Kartogenin
MIA	Monoiodoacetate
Micro-CT	Micro-computed tomography
MMP-13	Matrix metallopeptidase 13
MSCs	Mesenchymal stem cells
MSNs	Mesoporous silica nanoparticles
MMT	Medial meniscal transection
NPs	Nanoparticles
NSAIDs	Non-steroidal anti-inflammatory drugs
PBS	Phosphate buffered saline
PEGDA	Poly (ethylene glycol) diacrylate
PLA	Poly(D,L)-lactide
PLGA	Poly(lactic-coglycolic) acid
pNiPAM	poly(N-isopropylacrylamide)
PRISMA	Preferred Reporting Items for Systematic Reviews and Meta-Analyses
PTOA	Post-traumatic osteoarthritis
OA	Osteoarthritis
OARSI	Osteoarthritis Research Society International
RT-PCR	Real-time polymerase chain reaction
RUNX2	Runt-related transcription factor 2
SYRCLE	Systematic Review Centre for Laboratory Animal Experimentation
SOX9	SRY-box transcription factor 9
TCA	triamcinolone acetonide
TePNs	TCA-encapsulated polymeric nanoparticles
TNF-α	Tumor necrosis factor α
VEGF	Vascular endothelial growth factor
